# 11-[(*E*)-4-Bromo­benzyl­idene]-8-(4-bromo­phen­yl)-14-hy­droxy-3,13-diaza­hepta­cyclo­[13.7.1.1^9,13^.0^2,9^.0^2,14^.0^3,7^.0^19,23^]tetra­cosa-1(22),15,17,19(23),20-pentaen-10-one

**DOI:** 10.1107/S1600536810042091

**Published:** 2010-10-23

**Authors:** Raju Suresh Kumar, Hasnah Osman, Mohamed Ashraf Ali, Madhukar Hemamalini, Hoong-Kun Fun

**Affiliations:** a School of Chemical Sciences, Universiti Sains Malaysia, 11800 USM, Penang, Malaysia; b Institute for Research in Molecular Medicine, Universiti Sains Malaysia, 11800 USM, Penang, Malaysia; c X-ray Crystallography Unit, School of Physics, Universiti Sains Malaysia, 11800 USM, Penang, Malaysia

## Abstract

In the title compound, C_35_H_28_Br_2_N_2_O_2_, the piperidone ring adopts a chair conformation and the five-membered ring of the pyrrolidine ring adopts an envelope conformation. The naphthalene ring system makes dihedral angles of 37.12 (8) and 50.62 (9)° with the terminal bromo-substituted benzene rings. The dihedral angle between the two bromo-substituted benzene rings is 72.54 (10)°. In the crystal, adjacent mol­ecules are connected by a pair of inter­molecular C—H⋯O hydrogen bonds, forming an inversion dimer. An intra­molecular O—H⋯N hydrogen bond is also present.

## Related literature

For details of cyclo­addition, see: Babu & Raghunathan (2007 ▶); Boruah *et al.* (2007 ▶); Dondas *et al.* (2004 ▶); Hong *et al.* (2007 ▶); Karthikeyan *et al.* (2007 ▶); Liddell (1998 ▶); Ramesh *et al.* (2007 ▶). For puckering parameters, see: Cremer & Pople (1975 ▶). For the stability of the temperature controller used in the data collection, see: Cosier & Glazer (1986 ▶). 
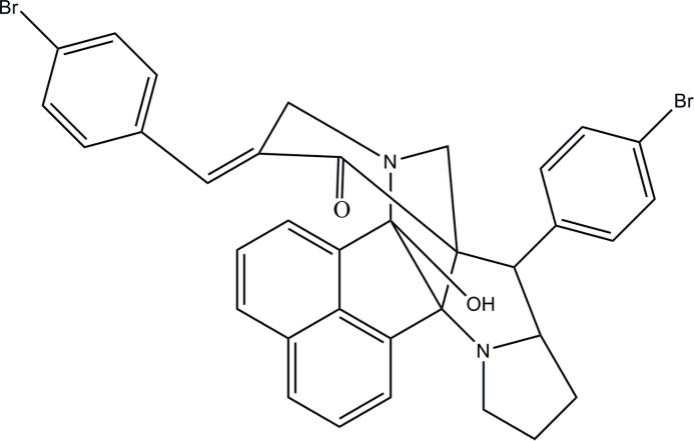


## Experimental

### 

#### Crystal data

C_35_H_28_Br_2_N_2_O_2_
*M* *_r_* = 668.41
Triclinic, 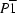

*a* = 8.4833 (10) Å
*b* = 11.8334 (13) Å
*c* = 14.8942 (17) Å
α = 79.868 (2)°
β = 80.705 (2)°
γ = 77.359 (2)°
*V* = 1424.4 (3) Å^3^
*Z* = 2
Mo *K*α radiation
μ = 2.88 mm^−1^
*T* = 100 K
0.44 × 0.17 × 0.16 mm

#### Data collection

Bruker APEXII DUO CCD area-detector diffractometer
Absorption correction: multi-scan (*SADABS*; Bruker, 2009 ▶) *T*_min_ = 0.364, *T*_max_ = 0.657
14706 measured reflections
6459 independent reflections
5384 reflections with *I* > 2σ(*I*)
*R*_int_ = 0.030

#### Refinement

*R*[*F* ^2^ > 2σ(*F* ^2^)] = 0.028
*wR*(*F* ^2^) = 0.077
*S* = 1.07
6459 reflections
374 parameters
H atoms treated by a mixture of independent and constrained refinement
Δρ_max_ = 0.45 e Å^−3^
Δρ_min_ = −0.48 e Å^−3^

### 

Data collection: *APEX2* (Bruker, 2009 ▶); cell refinement: *SAINT* (Bruker, 2009 ▶); data reduction: *SAINT*; program(s) used to solve structure: *SHELXTL* (Sheldrick, 2008 ▶); program(s) used to refine structure: *SHELXTL*; molecular graphics: *SHELXTL*; software used to prepare material for publication: *SHELXTL* and *PLATON* (Spek, 2009 ▶).

## Supplementary Material

Crystal structure: contains datablocks global, I. DOI: 10.1107/S1600536810042091/is2614sup1.cif

Structure factors: contains datablocks I. DOI: 10.1107/S1600536810042091/is2614Isup2.hkl

Additional supplementary materials:  crystallographic information; 3D view; checkCIF report

## Figures and Tables

**Table 1:** Hydrogen-bond geometry (Å, °)

*D*—H⋯*A*	*D*—H	H⋯*A*	*D*⋯*A*	*D*—H⋯*A*
O2—H1*O*2⋯N2	0.81 (3)	2.01 (2)	2.578 (2)	127 (3)
C20—H20*A*⋯O2^i^	0.98	2.46	3.130 (3)	125

Symmetry code: (i) 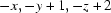.

**Fig. 1.**
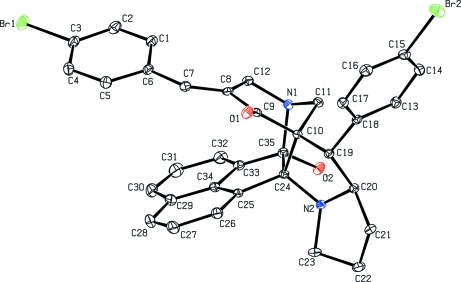
The asymmetric unit of the title compound, showing 30% probability displacement ellipsoids and the atom-numbering scheme (H atoms are omitted for clarity).

**Fig. 2.**
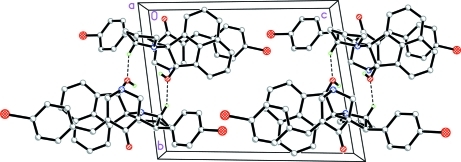
The crystal packing of the title compound, showing hydrogen-bonded (dashed lines) dimers. H atoms are not involving the hydrogen bond interactions are omitted for clarity.

## supplementary crystallographic information

### Comment

1,3-Dipolar cycloadditions form a subject of intensive research in organic synthesis in view of their great synthetic potential (Karthikeyan *et al.*, 2007; Hong *et al.*, 2007). In particular, the cycloaddition of nonstabilized azomethine ylides with olefins represents one of the most convergent approaches for the construction of five membered heterocycles (Dondas *et al.*, 2004; Boruah *et al.*, 2007). Acenaphthenequinone is a versatile precursor for azomethine ylide cycloaddition as it reacts with various l-amino acids generating reactive 1,3-dipoles (Babu & Raghunathan, 2007; Ramesh *et al.*, 2007). The pyrrolidine substructure occurs in many natural products of potential use in medicine and agriculture (Liddell, 1998).

The molecular structure of the title compound is shown in Fig. 1. The piperidine (N1/C8–C12) ring adopts a chair conformation [Q = 0.611 (2) Å, Θ = 139.77 (19) °, φ = 241.0 (3) ° ; Cremer & Pople, 1975]. The pyrrolidine ring (N2/C20–C23) adopts an envelope conformation [puckering parameters Q = 0.385 (2) Å, Θ = 112.7 (3) °]. The naphthalene (C25–34) ring makes dihedral angles of 37.12 (8)° and 50.62 (9)° with the terminal bromo-substituted benzene (C1–C6) and (C13–C18) rings. The dihedral angle between the two bromo-substituted benzene (C1–C6) and (C13–C18) rings is 72.54 (10)°.

In the crystal packing (Fig. 2), adjacent molecules are connected by intermolecular C20—H20A···O2 hydrogen bonds, forming dimers arranged in sheets parallel to the *bc*-plane. An intramolecular O—H···N hydrogen bond is also present.

### Experimental

A mixture of 3,5-bis[(*E*)-(4-bromophenyl)methylidene]tetrahydro-4(1*H*)-pyridinone (0.100 g, 0.231 mmol), acenaphthenequinone (0.042 g, 0.231 mmol) and proline (0.027 g, 0.231 mmol) were dissolved in methanol (5 ml) and refluxed for 30 minutes. After completion of the reaction as evident from TLC, the mixture was poured into water (50 ml). The precipitated solid was filtered and washed with water to afford the product which was recrystallised from ethyl acetate to reveal the title compound as colourless crystals.

### Refinement

The hydroxyl H atom H1O2 was located from a difference Fourier map and refined freely. The remaining H atoms were positioned geometrically [C—H = 0.93 or 0.97 Å] and were refined using a riding model, with *U*_iso_(H) = 1.2*U*_eq_(C).

### Crystal data

**Table:**

C_35_H_28_Br_2_N_2_O_2_	*Z* = 2
*M**_r_* = 668.41	*F*(000) = 676
Triclinic, *P*1	*D*_x_ = 1.559 Mg m^−^^3^
Hall symbol: -P 1	Mo *K*α radiation, λ = 0.71073 Å
*a* = 8.4833 (10) Å	Cell parameters from 6649 reflections
*b* = 11.8334 (13) Å	θ = 2.4–28.4°
*c* = 14.8942 (17) Å	µ = 2.88 mm^−^^1^
α = 79.868 (2)°	*T* = 100 K
β = 80.705 (2)°	Block, colourless
γ = 77.359 (2)°	0.44 × 0.17 × 0.16 mm
*V* = 1424.4 (3) Å^3^	

### Data collection

**Table:**

Bruker APEXII DUO CCD area-detector diffractometer	6459 independent reflections
Radiation source: fine-focus sealed tube	5384 reflections with *I* > 2σ(*I*)
graphite	*R*_int_ = 0.030
φ and ω scans	θ_max_ = 27.5°, θ_min_ = 2.1°
Absorption correction: multi-scan (*SADABS*; Bruker, 2009)	*h* = −11→10
*T*_min_ = 0.364, *T*_max_ = 0.657	*k* = −15→15
14706 measured reflections	*l* = −19→19

### Refinement

**Table:**

Refinement on *F*^2^	Primary atom site location: structure-invariant direct methods
Least-squares matrix: full	Secondary atom site location: difference Fourier map
*R*[*F*^2^ > 2σ(*F*^2^)] = 0.028	Hydrogen site location: inferred from neighbouring sites
*wR*(*F*^2^) = 0.077	H atoms treated by a mixture of independent and constrained refinement
*S* = 1.07	*w* = 1/[σ^2^(*F*_o_^2^) + (0.030*P*)^2^ + 0.5557*P*] where *P* = (*F*_o_^2^ + 2*F*_c_^2^)/3
6459 reflections	(Δ/σ)_max_ = 0.003
374 parameters	Δρ_max_ = 0.45 e Å^−^^3^
0 restraints	Δρ_min_ = −0.48 e Å^−^^3^

### Special details

**Table:**

Experimental. The crystal was placed in the cold stream of an Oxford Cryosystems Cobra open-flow nitrogen cryostat (Cosier & Glazer, 1986) operating at 100.0 (1) K.
Geometry. All s.u.'s (except the s.u. in the dihedral angle between two l.s. planes) are estimated using the full covariance matrix. The cell s.u.'s are taken into account individually in the estimation of s.u.'s in distances, angles and torsion angles; correlations between s.u.'s in cell parameters are only used when they are defined by crystal symmetry. An approximate (isotropic) treatment of cell s.u.'s is used for estimating s.u.'s involving l.s. planes.
Refinement. Refinement of F^2^ against ALL reflections. The weighted R-factor wR and goodness of fit S are based on F^2^, conventional R-factors R are based on F, with F set to zero for negative F^2^. The threshold expression of F^2^ > 2σ(F^2^) is used only for calculating R-factors(gt) etc. and is not relevant to the choice of reflections for refinement. R-factors based on F^2^ are statistically about twice as large as those based on F, and R- factors based on ALL data will be even larger.

### Fractional atomic coordinates and isotropic or equivalent isotropic displacement parameters (Å^2^)

**Table:**

	*x*	*y*	*z*	*U*_iso_*/*U*_eq_	
Br1	0.95912 (3)	0.70022 (2)	0.375928 (15)	0.03029 (7)	
Br2	0.50038 (3)	0.80345 (2)	1.346623 (16)	0.03079 (8)	
O1	0.36372 (17)	0.90896 (12)	0.88975 (10)	0.0203 (3)	
O2	0.11709 (19)	0.50546 (13)	0.90383 (10)	0.0213 (3)	
N1	0.3708 (2)	0.56045 (14)	0.88753 (11)	0.0159 (3)	
N2	−0.02666 (19)	0.70855 (14)	0.94557 (11)	0.0157 (3)	
C1	0.7446 (3)	0.66951 (19)	0.64742 (15)	0.0225 (4)	
H1A	0.7501	0.6124	0.6990	0.027*	
C2	0.8377 (3)	0.64507 (19)	0.56501 (15)	0.0254 (5)	
H2A	0.9044	0.5720	0.5610	0.030*	
C3	0.8299 (3)	0.7308 (2)	0.48903 (14)	0.0228 (4)	
C4	0.7332 (3)	0.84159 (19)	0.49339 (14)	0.0225 (4)	
H4A	0.7310	0.8990	0.4419	0.027*	
C5	0.6406 (3)	0.86453 (18)	0.57587 (14)	0.0213 (4)	
H5A	0.5758	0.9384	0.5794	0.026*	
C6	0.6419 (2)	0.77957 (18)	0.65411 (13)	0.0182 (4)	
C7	0.5367 (2)	0.81193 (17)	0.73743 (13)	0.0174 (4)	
H7A	0.5086	0.8919	0.7400	0.021*	
C8	0.4743 (2)	0.74428 (17)	0.81089 (13)	0.0157 (4)	
C9	0.3618 (2)	0.80907 (17)	0.88225 (13)	0.0150 (4)	
C10	0.2428 (2)	0.73992 (16)	0.94201 (13)	0.0136 (4)	
C11	0.3388 (2)	0.61511 (16)	0.97163 (13)	0.0158 (4)	
H11A	0.4399	0.6181	0.9929	0.019*	
H11B	0.2746	0.5724	1.0203	0.019*	
C12	0.4976 (2)	0.61138 (17)	0.82450 (13)	0.0168 (4)	
H12A	0.5039	0.5869	0.7650	0.020*	
H12B	0.6014	0.5787	0.8472	0.020*	
C13	0.2392 (2)	0.70348 (18)	1.17155 (14)	0.0192 (4)	
H13A	0.1953	0.6386	1.1697	0.023*	
C14	0.3250 (3)	0.70395 (19)	1.24397 (14)	0.0217 (4)	
H14A	0.3397	0.6396	1.2898	0.026*	
C15	0.3874 (2)	0.80129 (18)	1.24641 (14)	0.0201 (4)	
C16	0.3701 (3)	0.89758 (19)	1.17861 (14)	0.0228 (4)	
H16A	0.4137	0.9624	1.1810	0.027*	
C17	0.2856 (2)	0.89479 (18)	1.10674 (14)	0.0189 (4)	
H17A	0.2737	0.9589	1.0605	0.023*	
C18	0.2184 (2)	0.79958 (16)	1.10161 (13)	0.0151 (4)	
C19	0.1330 (2)	0.80060 (16)	1.01924 (13)	0.0145 (4)	
H19A	0.0937	0.8824	0.9940	0.017*	
C20	−0.0118 (2)	0.73723 (17)	1.03739 (13)	0.0157 (4)	
H20A	0.0135	0.6647	1.0802	0.019*	
C21	−0.1792 (2)	0.80658 (19)	1.07021 (14)	0.0216 (4)	
H21A	−0.1806	0.8900	1.0586	0.026*	
H21B	−0.2099	0.7836	1.1354	0.026*	
C22	−0.2933 (3)	0.7743 (2)	1.01281 (15)	0.0237 (4)	
H22A	−0.3252	0.7005	1.0398	0.028*	
H22B	−0.3904	0.8348	1.0072	0.028*	
C23	−0.1894 (2)	0.76447 (19)	0.91974 (15)	0.0218 (4)	
H23A	−0.2285	0.7163	0.8852	0.026*	
H23B	−0.1884	0.8410	0.8836	0.026*	
C24	0.1223 (2)	0.71519 (16)	0.88158 (13)	0.0137 (4)	
C25	0.1053 (2)	0.78952 (17)	0.78823 (13)	0.0165 (4)	
C26	0.0509 (3)	0.90806 (18)	0.76295 (15)	0.0220 (4)	
H26A	0.0155	0.9579	0.8071	0.026*	
C27	0.0505 (3)	0.9519 (2)	0.66793 (17)	0.0302 (5)	
H27A	0.0114	1.0313	0.6507	0.036*	
C28	0.1049 (3)	0.8824 (2)	0.60051 (16)	0.0333 (5)	
H28A	0.1034	0.9152	0.5391	0.040*	
C29	0.1642 (3)	0.7598 (2)	0.62403 (15)	0.0253 (5)	
C30	0.2261 (3)	0.6766 (2)	0.56313 (15)	0.0330 (6)	
H30A	0.2332	0.7008	0.5000	0.040*	
C31	0.2762 (3)	0.5598 (2)	0.59660 (16)	0.0326 (6)	
H31A	0.3143	0.5063	0.5554	0.039*	
C32	0.2708 (3)	0.5194 (2)	0.69231 (15)	0.0257 (5)	
H32A	0.3041	0.4404	0.7139	0.031*	
C33	0.2154 (2)	0.59901 (18)	0.75230 (14)	0.0186 (4)	
C34	0.1594 (2)	0.71801 (18)	0.71875 (14)	0.0187 (4)	
C35	0.2080 (2)	0.58723 (16)	0.85513 (13)	0.0156 (4)	
H1O2	0.035 (4)	0.538 (2)	0.9321 (19)	0.037 (8)*	

### Atomic displacement parameters (Å^2^)

**Table:**

	*U*^11^	*U*^22^	*U*^33^	*U*^12^	*U*^13^	*U*^23^
Br1	0.02851 (13)	0.03823 (14)	0.02268 (12)	−0.00510 (10)	0.00563 (9)	−0.01058 (9)
Br2	0.03305 (14)	0.03556 (14)	0.02841 (13)	−0.00501 (10)	−0.01459 (10)	−0.00954 (10)
O1	0.0203 (8)	0.0184 (7)	0.0235 (7)	−0.0077 (6)	0.0010 (6)	−0.0048 (6)
O2	0.0213 (8)	0.0153 (7)	0.0275 (8)	−0.0092 (6)	0.0025 (6)	−0.0015 (6)
N1	0.0155 (8)	0.0147 (8)	0.0164 (8)	−0.0019 (6)	−0.0024 (6)	−0.0002 (6)
N2	0.0117 (8)	0.0177 (8)	0.0184 (8)	−0.0053 (6)	−0.0026 (6)	−0.0006 (6)
C1	0.0177 (11)	0.0235 (11)	0.0230 (10)	−0.0020 (8)	0.0011 (8)	−0.0004 (8)
C2	0.0218 (11)	0.0233 (11)	0.0289 (11)	−0.0033 (9)	0.0038 (9)	−0.0057 (9)
C3	0.0185 (10)	0.0330 (12)	0.0182 (10)	−0.0077 (9)	0.0028 (8)	−0.0084 (9)
C4	0.0254 (11)	0.0249 (11)	0.0177 (10)	−0.0079 (9)	−0.0022 (8)	−0.0017 (8)
C5	0.0228 (11)	0.0196 (10)	0.0216 (10)	−0.0051 (8)	−0.0021 (8)	−0.0031 (8)
C6	0.0154 (10)	0.0223 (10)	0.0179 (10)	−0.0068 (8)	−0.0007 (8)	−0.0032 (8)
C7	0.0161 (10)	0.0180 (9)	0.0192 (10)	−0.0049 (8)	−0.0027 (8)	−0.0032 (8)
C8	0.0095 (9)	0.0199 (10)	0.0182 (9)	−0.0022 (7)	−0.0028 (7)	−0.0041 (8)
C9	0.0129 (9)	0.0165 (9)	0.0161 (9)	−0.0025 (7)	−0.0050 (7)	−0.0011 (7)
C10	0.0118 (9)	0.0146 (9)	0.0142 (9)	−0.0021 (7)	−0.0031 (7)	−0.0014 (7)
C11	0.0156 (10)	0.0142 (9)	0.0164 (9)	−0.0012 (7)	−0.0037 (7)	−0.0002 (7)
C12	0.0136 (10)	0.0169 (9)	0.0194 (9)	−0.0018 (7)	−0.0022 (7)	−0.0029 (8)
C13	0.0195 (10)	0.0199 (10)	0.0189 (10)	−0.0065 (8)	−0.0011 (8)	−0.0032 (8)
C14	0.0222 (11)	0.0246 (11)	0.0166 (10)	−0.0020 (9)	−0.0030 (8)	−0.0010 (8)
C15	0.0162 (10)	0.0259 (11)	0.0195 (10)	−0.0018 (8)	−0.0032 (8)	−0.0090 (8)
C16	0.0216 (11)	0.0246 (11)	0.0250 (11)	−0.0071 (9)	−0.0034 (9)	−0.0076 (9)
C17	0.0181 (10)	0.0183 (10)	0.0201 (10)	−0.0042 (8)	−0.0012 (8)	−0.0027 (8)
C18	0.0119 (9)	0.0157 (9)	0.0165 (9)	−0.0011 (7)	0.0001 (7)	−0.0030 (7)
C19	0.0127 (9)	0.0128 (9)	0.0171 (9)	−0.0022 (7)	−0.0013 (7)	−0.0007 (7)
C20	0.0139 (10)	0.0157 (9)	0.0171 (9)	−0.0039 (8)	−0.0018 (7)	−0.0004 (7)
C21	0.0129 (10)	0.0273 (11)	0.0222 (10)	−0.0020 (8)	0.0011 (8)	−0.0026 (8)
C22	0.0135 (10)	0.0265 (11)	0.0298 (11)	−0.0034 (8)	−0.0023 (8)	−0.0021 (9)
C23	0.0127 (10)	0.0257 (11)	0.0282 (11)	−0.0029 (8)	−0.0063 (8)	−0.0046 (9)
C24	0.0120 (9)	0.0121 (9)	0.0172 (9)	−0.0037 (7)	−0.0031 (7)	0.0001 (7)
C25	0.0121 (9)	0.0200 (10)	0.0172 (9)	−0.0055 (8)	−0.0044 (7)	0.0029 (8)
C26	0.0199 (11)	0.0185 (10)	0.0263 (11)	−0.0045 (8)	−0.0043 (8)	0.0026 (8)
C27	0.0277 (13)	0.0243 (11)	0.0343 (13)	−0.0051 (10)	−0.0081 (10)	0.0109 (10)
C28	0.0298 (13)	0.0455 (15)	0.0213 (11)	−0.0106 (11)	−0.0075 (10)	0.0115 (10)
C29	0.0211 (11)	0.0355 (12)	0.0210 (10)	−0.0105 (9)	−0.0066 (9)	0.0009 (9)
C30	0.0293 (13)	0.0566 (16)	0.0163 (10)	−0.0149 (12)	−0.0037 (9)	−0.0050 (10)
C31	0.0305 (13)	0.0462 (15)	0.0266 (12)	−0.0114 (11)	−0.0012 (10)	−0.0172 (11)
C32	0.0251 (12)	0.0286 (12)	0.0270 (11)	−0.0082 (9)	−0.0037 (9)	−0.0095 (9)
C33	0.0143 (10)	0.0240 (10)	0.0194 (10)	−0.0072 (8)	−0.0029 (8)	−0.0031 (8)
C34	0.0165 (10)	0.0231 (10)	0.0185 (10)	−0.0081 (8)	−0.0060 (8)	0.0002 (8)
C35	0.0148 (10)	0.0139 (9)	0.0182 (9)	−0.0044 (7)	−0.0013 (7)	−0.0012 (7)

### Geometric parameters (Å, °)

**Table:**

Br1—C3	1.901 (2)	C15—C16	1.382 (3)
Br2—C15	1.9063 (19)	C16—C17	1.390 (3)
O1—C9	1.210 (2)	C16—H16A	0.9300
O2—C35	1.399 (2)	C17—C18	1.388 (3)
O2—H1O2	0.81 (3)	C17—H17A	0.9300
N1—C11	1.470 (2)	C18—C19	1.519 (3)
N1—C12	1.470 (3)	C19—C20	1.539 (3)
N1—C35	1.487 (2)	C19—H19A	0.9800
N2—C24	1.463 (2)	C20—C21	1.529 (3)
N2—C23	1.474 (2)	C20—H20A	0.9800
N2—C20	1.496 (2)	C21—C22	1.536 (3)
C1—C2	1.388 (3)	C21—H21A	0.9700
C1—C6	1.409 (3)	C21—H21B	0.9700
C1—H1A	0.9300	C22—C23	1.526 (3)
C2—C3	1.380 (3)	C22—H22A	0.9700
C2—H2A	0.9300	C22—H22B	0.9700
C3—C4	1.392 (3)	C23—H23A	0.9700
C4—C5	1.383 (3)	C23—H23B	0.9700
C4—H4A	0.9300	C24—C25	1.519 (3)
C5—C6	1.398 (3)	C24—C35	1.616 (3)
C5—H5A	0.9300	C25—C26	1.382 (3)
C6—C7	1.464 (3)	C25—C34	1.408 (3)
C7—C8	1.345 (3)	C26—C27	1.419 (3)
C7—H7A	0.9300	C26—H26A	0.9300
C8—C9	1.506 (3)	C27—C28	1.368 (4)
C8—C12	1.523 (3)	C27—H27A	0.9300
C9—C10	1.515 (3)	C28—C29	1.428 (3)
C10—C19	1.536 (2)	C28—H28A	0.9300
C10—C11	1.549 (3)	C29—C34	1.408 (3)
C10—C24	1.569 (2)	C29—C30	1.415 (3)
C11—H11A	0.9700	C30—C31	1.382 (4)
C11—H11B	0.9700	C30—H30A	0.9300
C12—H12A	0.9700	C31—C32	1.418 (3)
C12—H12B	0.9700	C31—H31A	0.9300
C13—C14	1.397 (3)	C32—C33	1.369 (3)
C13—C18	1.401 (3)	C32—H32A	0.9300
C13—H13A	0.9300	C33—C34	1.412 (3)
C14—C15	1.377 (3)	C33—C35	1.505 (3)
C14—H14A	0.9300		
			
C35—O2—H1O2	110 (2)	C18—C19—C20	116.38 (16)
C11—N1—C12	108.25 (15)	C10—C19—C20	101.97 (14)
C11—N1—C35	102.77 (14)	C18—C19—H19A	108.0
C12—N1—C35	115.10 (15)	C10—C19—H19A	108.0
C24—N2—C23	122.19 (16)	C20—C19—H19A	108.0
C24—N2—C20	111.43 (14)	N2—C20—C21	105.40 (15)
C23—N2—C20	109.51 (15)	N2—C20—C19	104.64 (15)
C2—C1—C6	121.0 (2)	C21—C20—C19	117.58 (16)
C2—C1—H1A	119.5	N2—C20—H20A	109.6
C6—C1—H1A	119.5	C21—C20—H20A	109.6
C3—C2—C1	119.1 (2)	C19—C20—H20A	109.6
C3—C2—H2A	120.5	C20—C21—C22	103.60 (16)
C1—C2—H2A	120.5	C20—C21—H21A	111.0
C2—C3—C4	121.69 (19)	C22—C21—H21A	111.0
C2—C3—Br1	119.80 (16)	C20—C21—H21B	111.0
C4—C3—Br1	118.47 (16)	C22—C21—H21B	111.0
C5—C4—C3	118.6 (2)	H21A—C21—H21B	109.0
C5—C4—H4A	120.7	C23—C22—C21	103.12 (16)
C3—C4—H4A	120.7	C23—C22—H22A	111.1
C4—C5—C6	121.70 (19)	C21—C22—H22A	111.1
C4—C5—H5A	119.1	C23—C22—H22B	111.1
C6—C5—H5A	119.1	C21—C22—H22B	111.1
C5—C6—C1	117.91 (18)	H22A—C22—H22B	109.1
C5—C6—C7	117.39 (18)	N2—C23—C22	102.76 (16)
C1—C6—C7	124.70 (19)	N2—C23—H23A	111.2
C8—C7—C6	130.21 (19)	C22—C23—H23A	111.2
C8—C7—H7A	114.9	N2—C23—H23B	111.2
C6—C7—H7A	114.9	C22—C23—H23B	111.2
C7—C8—C9	115.48 (17)	H23A—C23—H23B	109.1
C7—C8—C12	126.23 (17)	N2—C24—C25	117.47 (15)
C9—C8—C12	118.14 (17)	N2—C24—C10	103.75 (14)
O1—C9—C8	122.78 (18)	C25—C24—C10	118.48 (16)
O1—C9—C10	122.87 (17)	N2—C24—C35	110.44 (15)
C8—C9—C10	114.33 (16)	C25—C24—C35	102.85 (15)
C9—C10—C19	115.73 (15)	C10—C24—C35	102.89 (14)
C9—C10—C11	107.62 (15)	C26—C25—C34	118.66 (18)
C19—C10—C11	115.94 (16)	C26—C25—C24	131.91 (18)
C9—C10—C24	109.71 (15)	C34—C25—C24	109.42 (17)
C19—C10—C24	104.93 (15)	C25—C26—C27	118.3 (2)
C11—C10—C24	101.86 (14)	C25—C26—H26A	120.8
N1—C11—C10	103.98 (14)	C27—C26—H26A	120.8
N1—C11—H11A	111.0	C28—C27—C26	122.9 (2)
C10—C11—H11A	111.0	C28—C27—H27A	118.6
N1—C11—H11B	111.0	C26—C27—H27A	118.6
C10—C11—H11B	111.0	C27—C28—C29	120.3 (2)
H11A—C11—H11B	109.0	C27—C28—H28A	119.8
N1—C12—C8	116.06 (16)	C29—C28—H28A	119.8
N1—C12—H12A	108.3	C34—C29—C30	116.9 (2)
C8—C12—H12A	108.3	C34—C29—C28	115.7 (2)
N1—C12—H12B	108.3	C30—C29—C28	127.4 (2)
C8—C12—H12B	108.3	C31—C30—C29	120.6 (2)
H12A—C12—H12B	107.4	C31—C30—H30A	119.7
C14—C13—C18	120.83 (19)	C29—C30—H30A	119.7
C14—C13—H13A	119.6	C30—C31—C32	121.6 (2)
C18—C13—H13A	119.6	C30—C31—H31A	119.2
C15—C14—C13	118.9 (2)	C32—C31—H31A	119.2
C15—C14—H14A	120.5	C33—C32—C31	118.6 (2)
C13—C14—H14A	120.5	C33—C32—H32A	120.7
C14—C15—C16	122.01 (18)	C31—C32—H32A	120.7
C14—C15—Br2	118.98 (16)	C32—C33—C34	120.08 (19)
C16—C15—Br2	119.01 (15)	C32—C33—C35	131.8 (2)
C15—C16—C17	118.09 (19)	C34—C33—C35	108.01 (16)
C15—C16—H16A	121.0	C29—C34—C25	124.07 (19)
C17—C16—H16A	121.0	C29—C34—C33	122.07 (19)
C18—C17—C16	122.2 (2)	C25—C34—C33	113.82 (17)
C18—C17—H17A	118.9	O2—C35—N1	108.74 (15)
C16—C17—H17A	118.9	O2—C35—C33	113.94 (15)
C17—C18—C13	117.91 (17)	N1—C35—C33	113.66 (16)
C17—C18—C19	119.40 (17)	O2—C35—C24	108.79 (15)
C13—C18—C19	122.62 (17)	N1—C35—C24	105.65 (14)
C18—C19—C10	113.92 (15)	C33—C35—C24	105.59 (15)
			
C6—C1—C2—C3	0.6 (3)	C23—N2—C24—C25	4.1 (2)
C1—C2—C3—C4	1.1 (3)	C20—N2—C24—C25	−128.02 (17)
C1—C2—C3—Br1	178.94 (16)	C23—N2—C24—C10	137.01 (17)
C2—C3—C4—C5	−1.3 (3)	C20—N2—C24—C10	4.87 (19)
Br1—C3—C4—C5	−179.23 (15)	C23—N2—C24—C35	−113.35 (18)
C3—C4—C5—C6	−0.1 (3)	C20—N2—C24—C35	114.51 (16)
C4—C5—C6—C1	1.7 (3)	C9—C10—C24—N2	−150.70 (15)
C4—C5—C6—C7	−178.66 (19)	C19—C10—C24—N2	−25.76 (18)
C2—C1—C6—C5	−2.0 (3)	C11—C10—C24—N2	95.48 (16)
C2—C1—C6—C7	178.4 (2)	C9—C10—C24—C25	−18.4 (2)
C5—C6—C7—C8	157.1 (2)	C19—C10—C24—C25	106.54 (18)
C1—C6—C7—C8	−23.3 (3)	C11—C10—C24—C25	−132.22 (17)
C6—C7—C8—C9	−176.58 (18)	C9—C10—C24—C35	94.16 (17)
C6—C7—C8—C12	−1.1 (3)	C19—C10—C24—C35	−140.89 (15)
C7—C8—C9—O1	−21.9 (3)	C11—C10—C24—C35	−19.66 (18)
C12—C8—C9—O1	162.27 (18)	N2—C24—C25—C26	63.1 (3)
C7—C8—C9—C10	156.46 (17)	C10—C24—C25—C26	−62.8 (3)
C12—C8—C9—C10	−19.4 (2)	C35—C24—C25—C26	−175.4 (2)
O1—C9—C10—C19	−5.1 (3)	N2—C24—C25—C34	−117.82 (18)
C8—C9—C10—C19	176.55 (15)	C10—C24—C25—C34	116.24 (18)
O1—C9—C10—C11	−136.63 (19)	C35—C24—C25—C34	3.7 (2)
C8—C9—C10—C11	45.05 (19)	C34—C25—C26—C27	0.7 (3)
O1—C9—C10—C24	113.3 (2)	C24—C25—C26—C27	179.7 (2)
C8—C9—C10—C24	−65.00 (19)	C25—C26—C27—C28	−1.6 (3)
C12—N1—C11—C10	73.46 (18)	C26—C27—C28—C29	0.7 (4)
C35—N1—C11—C10	−48.73 (18)	C27—C28—C29—C34	1.0 (3)
C9—C10—C11—N1	−72.90 (17)	C27—C28—C29—C30	−179.3 (2)
C19—C10—C11—N1	155.71 (15)	C34—C29—C30—C31	1.1 (3)
C24—C10—C11—N1	42.46 (18)	C28—C29—C30—C31	−178.6 (2)
C11—N1—C12—C8	−47.2 (2)	C29—C30—C31—C32	−1.3 (4)
C35—N1—C12—C8	67.1 (2)	C30—C31—C32—C33	−0.5 (3)
C7—C8—C12—N1	−155.72 (18)	C31—C32—C33—C34	2.4 (3)
C9—C8—C12—N1	19.6 (2)	C31—C32—C33—C35	−174.0 (2)
C18—C13—C14—C15	0.8 (3)	C30—C29—C34—C25	178.4 (2)
C13—C14—C15—C16	−1.1 (3)	C28—C29—C34—C25	−1.8 (3)
C13—C14—C15—Br2	178.65 (15)	C30—C29—C34—C33	0.8 (3)
C14—C15—C16—C17	0.5 (3)	C28—C29—C34—C33	−179.4 (2)
Br2—C15—C16—C17	−179.23 (15)	C26—C25—C34—C29	1.0 (3)
C15—C16—C17—C18	0.4 (3)	C24—C25—C34—C29	−178.22 (18)
C16—C17—C18—C13	−0.6 (3)	C26—C25—C34—C33	178.72 (18)
C16—C17—C18—C19	−177.76 (18)	C24—C25—C34—C33	−0.5 (2)
C14—C13—C18—C17	0.0 (3)	C32—C33—C34—C29	−2.6 (3)
C14—C13—C18—C19	177.05 (18)	C35—C33—C34—C29	174.52 (18)
C17—C18—C19—C10	94.5 (2)	C32—C33—C34—C25	179.59 (19)
C13—C18—C19—C10	−82.5 (2)	C35—C33—C34—C25	−3.3 (2)
C17—C18—C19—C20	−147.24 (18)	C11—N1—C35—O2	−81.90 (17)
C13—C18—C19—C20	35.7 (3)	C12—N1—C35—O2	160.66 (15)
C9—C10—C19—C18	−76.7 (2)	C11—N1—C35—C33	150.04 (16)
C11—C10—C19—C18	50.8 (2)	C12—N1—C35—C33	32.6 (2)
C24—C10—C19—C18	162.24 (15)	C11—N1—C35—C24	34.74 (18)
C9—C10—C19—C20	157.07 (15)	C12—N1—C35—C24	−82.70 (18)
C11—C10—C19—C20	−75.47 (19)	C32—C33—C35—O2	−58.7 (3)
C24—C10—C19—C20	36.01 (18)	C34—C33—C35—O2	124.68 (18)
C24—N2—C20—C21	142.38 (16)	C32—C33—C35—N1	66.7 (3)
C23—N2—C20—C21	4.1 (2)	C34—C33—C35—N1	−109.99 (18)
C24—N2—C20—C19	17.7 (2)	C32—C33—C35—C24	−178.0 (2)
C23—N2—C20—C19	−120.52 (17)	C34—C33—C35—C24	5.4 (2)
C18—C19—C20—N2	−157.36 (15)	N2—C24—C35—O2	−1.9 (2)
C10—C19—C20—N2	−32.76 (18)	C25—C24—C35—O2	−128.07 (16)
C18—C19—C20—C21	86.1 (2)	C10—C24—C35—O2	108.29 (16)
C10—C19—C20—C21	−149.26 (17)	N2—C24—C35—N1	−118.52 (16)
N2—C20—C21—C22	20.2 (2)	C25—C24—C35—N1	115.33 (16)
C19—C20—C21—C22	136.26 (18)	C10—C24—C35—N1	−8.31 (19)
C20—C21—C22—C23	−36.4 (2)	N2—C24—C35—C33	120.76 (16)
C24—N2—C23—C22	−159.72 (16)	C25—C24—C35—C33	−5.39 (19)
C20—N2—C23—C22	−26.8 (2)	C10—C24—C35—C33	−129.03 (16)
C21—C22—C23—N2	38.7 (2)		

### Hydrogen-bond geometry (Å, °)

**Table:**

*D*—H···*A*	*D*—H	H···*A*	*D*···*A*	*D*—H···*A*
O2—H1O2···N2	0.81 (3)	2.01 (2)	2.578 (2)	127 (3)
C20—H20A···O2^i^	0.98	2.46	3.130 (3)	125

Symmetry codes: (i) −*x*, −*y*+1, −*z*+2.
